# Inequity in healthcare needs, health service use and financial burden of medical expenditures in China: results from a consecutive household monitoring study in Jiangsu Province

**DOI:** 10.1186/s12913-019-4796-4

**Published:** 2019-12-16

**Authors:** Weixi Jiang, Xiaolin Xu, Shenglan Tang, Ling Xu, Yaoguang Zhang, Chris Elbers, Frank Cobelens, Lijing Yan

**Affiliations:** 1 Global Health Research Center, Duke Kunshan University, Kunshan, 215316 China; 2 School of Public Health, The University of Queensland, Brisbane, 4006 Australia; 3 Duke Global Health Institute, Duke University, Durham, NC 27710 USA; 4 Health human Resources Development Center, National Health Commission, Beijing, 100810 China; 5 Center for Health Statistics and Information, National Health Commission, Beijing, 100810 China; 6 School of Business and Economics, Vrije Universiteit Amsterdam, 1081 HV Amsterdam, Netherlands; 7 Department of Global Health and Amsterdam Institute for Global Health and Development, Amsterdam University Medical Centers, 1105 BP Amsterdam, Netherlands; 8 Global Health Research Center, Duke Kunshan University, No. 8 Duke Avenue, Kunshan, 215316 China

**Keywords:** Equity in healthcare, Healthcare needs, Health service use, Healthcare financing

## Abstract

**Background:**

Although public medical insurance covers over 95% of the population in China, disparities in health service use and out-of-pocket (OOP) health expenditure across income groups are still widely observed. This study aims to investigate the socio-economic disparities in perceived healthcare needs, informal care, formal care and payment for healthcare and explore their equity implication.

**Methods:**

We assessed healthcare needs, service use and payment in 400 households in rural and urban areas in Jiangsu, China, and included only the adult sample (*N* = 925). One baseline survey and 10 follow-up surveys were conducted during the 7-month monitoring period, and the Affordability Ladder Program (ALP) framework was adopted for data analysis. Negative binomial/zero-inflated negative binomial and logit regression models were used to explore factors associated with perceived needs of care and with the use of self-treatment, outpatient and inpatient care respectively. Two-part model and logit regression modeling were conducted to explore factors associated with OOP health expenditure and with the likelihood of incurring catastrophic health expenditure (CHE).

**Results:**

After adjusting for covariates, rural residence was significantly associated with more perceived healthcare needs, more self-treatment, higher probability of using outpatient and inpatient service, more OOP health expenditure and higher likelihood of incurring catastrophic expenditure (*P* < 0.05). Compared to the Urban Employee Basic Medical Insurance (UEBMI), enrollment in the New Rural Cooperative Medical Scheme (NRCMS) or in the Urban Resident Basic Medical Insurance (URBMI) was correlated with lower probability of ever using outpatient services, but with more outpatient visits when people were at risk of using outpatient service (*P* < 0.05). NRCMS/URBMI enrollment was also associated with higher likelihood of incurring CHE compared to UEBMI enrollment (OR = 2.02, *P* < 0.05); in stratified analysis of the rural and urban sample this effect was only significant for the rural population.

**Conclusions:**

The rural population in Jiangsu perceived more healthcare needs, had a higher probability of using both informal and formal healthcare services, and had more OOP health expenditure and a higher likelihood of incurring CHE. The inequity mainly exists in health care financing, and may be partially addressed through improving the benefit packages of NRCMS/URBMI.

## Background

The Chinese health care system has experienced rapid changes along with socio-economic reform. On the supply side, the government has in recent years been investing in health care infrastructure, especially in primary health care institutions [1]. On the demand side, three public health insurance schemes, the Urban Employee Basic Medical Insurance (UEBMI), the Urban Resident Basic Medical Insurance (URBMI) and the New Rural Cooperative Medical Scheme (NRCMS), were gradually established since late 1990s. Currently over 95% of the Chinese population are covered by these three public insurance schemes, leaving less than 5% not covered by any insurance scheme, and medical services are mostly financed through the co-payment mechanism of the insurance schemes [2]. Inpatient medical services are covered with reimbursement rates ranging from 50 to 90% for different schemes at different levels of hospitals, but the reimbursement rates for outpatient services are still rather low for URBMI and NRCMS [3, 4].

Many studies have assessed equity in health care utilization and financing in terms of insurance types and income. Recent studies on the three public insurance schemes generally show that they improved people’s access to formal care, including both outpatient and inpatient services, and narrowed the gaps in service use across income groups with the expansion of insurance coverage [3, 5–8]. Nevertheless, one report from the World Bank showed that the separation in the management of the financing and benefit packages of health insurance schemes across insurance types and regions weakened the risk-pooling effect, and caused inequity problems [9]. The high proportion of out-of-pocket (OOP) health expenditure also indicated obstacles to accessing health care and high financial burden of health care [10–12]. For each type of insurance specifically, research on URBMI and UEBMI reveals that these schemes benefited patients from higher income groups more, as poorer patients were less likely to use expensive care, thus receiving less reimbursement than the well-off, and the urban insurance systems failed to reduce the OOP health expenditure for the disadvantaged group [6, 13, 14]. For NRCMS studies show that the expansion of NRCMS narrowed the gaps in inpatient service use and encouraged poorer patients to seek informal and preventive care. However they also showed that the care utilization pattern under NRCMS was still pro-rich and that NRCMS did not lower health expenditure nor provide sufficient protection for the poor [7, 8, 15].

A rich literature also explored inequity in health care regarding a series of demographic and socio-economic factors. Several recent studies revealed that with the expansion of insurance coverage, the rural-urban disparities in health service utilization and reimbursement rate substantially narrowed in recent years, but still exist [16–18]. Rural patients enjoyed lower reimbursements and bore a high burden of medical expenditure with reference to their income level [17, 19]. Studies also showed how ethnic minority, income, education and insurance coverage may have a differential impact on the service use pattern of rural and urban populations [20, 21]. Nevertheless, there is a lack of systematic analysis on how demographic and socio-economic factors would impact on the whole health seeking process under the current health care system, starting from health care needs to payment for services for both rural and urban population.

In this study we referred to the Affordability Ladder Program (ALP) which provides a holistic approach to examining equity in the health care system from the demand-side perspective through step-by-step analysis, taking into account the perceived health care needs, informal care, formal care and payment for health care [22]. Several studies have used this framework to explore access to and payment for healthcare services in other countries [22–25]. This framework also allowed the exploration of the potential differential or synergistic impact of a certain factor on health equity in each step of the entire health care seeking path, which was not possible in most previous studies and were the main original contribution of our research. This study was located in Jiangsu, a relatively well-developed province in eastern China with a per capita GDP of $14,000 in 2015, where over 95% of the population were covered by one of the three public health insurance schemes [26]. We focused on the equity implication of non-need factors for health care such as residence, income and insurance coverage [27], and explored how these factors may influence each ladder step of health care for the rural and urban population, respectively.

## Methods

### Sampling design

One urban district (Gusu) and one rural county (Jinhu) in Jiangsu were selected as study sites. Gusu and Jinhu are located in southern and northern Jiangsu respectively, and the GDP per capita (RMB 136,556) in Gusu is twice as in Jinhu (RMB 65,535). Disproportionate stratified sampling was applied with a sample size of 200 households in each site. A list of households with non-communicable disease (NCD) patients was obtained from the local health bureau, and 100 households were randomly selected from the list in each site. The other 100 sample households were randomly selected from the rest of the households in the study sites. As the sample size is relatively small, the households with NCD patients were over-sampled in order to increase the total events of service use. The project also aims to look at NCD management for which the results are yet to be published.

### Data collection

This study consisted of a baseline survey and 10 follow-up surveys over 7 consecutive months during 2015–2016, with the first 6 surveys conducted bi-weekly and the last 4-monthly (the first 6 surveys were in phase 1, the remaining 4 were in phase 2 when survey frequency was reduced with regard to respondents’ feedback that the surveys in phase 1 were too frequent; see Additional file 1 for the Questionnaire). One knowledgeable person from each household, usually the household head, signed informed consent before the interview, and answered on behalf of all members in the household. The baseline survey gathered participants’ basic demographic, socio-economic and health information, as well as inpatient service use and expenditure in the past year. Each follow-up survey contained 6 questionnaires concerning chronic disease management for different NCDs, emergent illness (including emergent conditions of NCDs), patients’ utilization of self-treatment, outpatient and inpatient services as well as medical costs and out-of-pocket payments.

The baseline survey and the last follow-up survey of phase 1 (6th) and phase 2 (10th) were conducted through face-to-face interviews by trained interviewers in the participants’ homes. For other follow-up surveys participants were asked to choose between: 1) filling survey questionnaires themselves; 2) call interviewers when disease/health service use occurred; 3) face-to-face interview at home; or 4) telephone interview for the follow-up survey. More than 90% of households chose face-to-face interview, a few opted for telephone interview and almost no households filled the survey questionnaires themselves or called interviewers.

All questionnaires were checked by supervisors of the interviewers before transferring to the investigators. For quality assurance, a 5% sample of the questionnaires were randomly selected and double-checked by investigators through telephone re-interview; the rate of concordance exceeded 95%. Data was double entered, and inconsistencies, outliers and missing values were also double-checked in order to ensure data quality. Datasets were encrypted in storage and de-identified during data analysis to protect participants’ confidentiality.

### Data analysis

We included only the adult population in the analysis as children usually do not make care seeking decisions themselves [28]. Descriptive analyses were conducted to examine the demographic and socio-economic characteristics of the overall sample as well as of the urban and rural sample separately. The chi-squared test and t-test were applied to test for significance of differences between the rural and the urban sample. Based on the ALP framework [22] we identified 6 key outcome variables of interests throughout the care-seeking path as shown in Table 1. The total of reporting emergent illness episodes, conducting self-treatment, outpatient service use, inpatient service use, and the total amount of OOP health expenditure were aggregated over the 7-month survey period. Catastrophic health expenditure was defined as total OOP health expenditure exceeding 10% of household income [29]. The data were analyzed using STATA 13.1(StataCorp, Texas, USA).

**Table 1:** Key outcome variables identified based on the ALP framework

Healthcare seeking path	Outcome variables
Perceived health care needs	1) number of self-reported emergent illness episodes
Health service use	
Informal care	2) self-treatment
Formal care	3) outpatient service use
	4) inpatient service use
Payment for health care	5) total OOP health expenditure
Financial burden of health care	6) catastrophic health expenditure (CHE) during the survey period

**Table 2:** Basic characteristics of study participants (%)

	Total sample	Gusu	Jinhu
		(urban)	(rural)
	*n* = 925	*n* = 463	*n* = 462
Gender			
male	51.2	47.08	49.6
Age			
average (sd)	54.5 (17.0)	53.5 (18.8)	55.5 (15.0)
18–29	10.7	12.3	9.1
30–59	45.2	41.3	49.1
> =60	44.1	46.4	41.8
Marriage			
married	85.4	85.3	85.5
Education			
Below primary	15.2	2.6	27.9
primary and junior high	48.7	41	56.3
senior high school and above	36.1	56.4	15.8
Employment			
employed	55.0	37.8	72.3
retired	27.1	50.8	3.3
unemployed	21.4	17	26.2
Health insurance			
UEBMI	40.5	73.4	7.6
URBMI	6.2	7.6	4.8
NRCMS	49	10.6	87.5
other and no insurance	4.3	8.4	0.2
Mean household income per capita (RMB)			
average (sd)	2135 (1395)	2807 (1462)	1462 (920)
With NCD			
yes	45.7	43.8	47.6
Self-reported emergent illness episodes			
mean (sd)	0.98 (1.66)	0.40 (1.12)	1.56 (1.90)
Total times of self-treatment			
mean (sd)	0.39 (0.94)	0.06 (0.37)	0.71 (1.19)
Total times of outpatient service			
mean (sd)	0.66 (1.34)	0.35 (0.96)	0.97 (1.57)
% inpatient service use	6.0	3.9	8.0
Total OOP health expenditure			
mean (sd)	594 (4266)	201 (1940)	991 (5697)
% CHE	16.8	6.5	27.1

Multivariate regression models were used to explore factors associated with each of these outcome variables of interests. As preliminary analyses showed that there were many “zeros” in the data (no emergent illness, no service use and no health expenditure), we considered standard Poisson/negative binomial (NB) models versus zero-inflated Poisson (ZIP) /negative binomial (ZINB) models for the first three outcomes which were count variables, and a generalized linear model (GLM) versus a two part model combining logit regression and GLM for the OOP health expenditure [30]. The zero-inflated models have two processes that separately model the likelihood of not being at risk of having the event (process 1, note that a positive coefficient or relative risk > 1 implies a lower probability of being at risk) and the total number of events given that one is at risk (process 2). The two-part model estimates the likelihood of incurring any OOP medical expenditure and the amount of expenditure if incurred in two steps [30]. As for model selection, we considered the Akaike Information Criterion (AIC) and the Bayesian Information Criterion (BIC) of each model first, and Vuong’s closeness test for ZINB vs. the standard negative binomial model if AIC and BIC preferred different models [31]. Based on these criteria we selected the ZINB model for the total episodes of self-reported emergent illness, the NB model for self-treatment, the ZINB model for outpatient service use and two-part models combining logit regression and GLM for OOP health expenditure (see Additional file 2: Table S1). As only 9 patients in our sample were admitted more than once, we coded inpatient service use as a binary variable of use/non-use. Logit regression was conducted to analyze factors associated with the likelihood of ever using inpatient services and incurring CHE.

As for the independent variables, we focused on the effects of factors reflecting socio-economic status (SES), including rural/urban residence, education level, income, employment and the status of health insurance, and adjusted for factors that may affect both these SES factors and the outcomes, including age, gender, marital status and presence of NCDs. Age, income and education level were treated as categorical variables in the regression models. As for insurance, we grouped people with the new cooperative medical scheme (NRCMS) together with those enrolled in urban resident basic medical insurance (URBMI), as they provided similar benefit packages and only 6% of the sample were enrolled in URBMI. The average income per capita was divided into 3 groups: the richest 33.3%, middle 33.3% and poorest 33.3% for the whole sample. Standard errors were adjusted for household clustering considering the intra-household correlation.

## Results

### Sample characteristics

Four-hundred households participated and completed the surveys, totaling 1057 people. We included the 925 adult participants in the analyses. Table 1 shows the demographic, socio-economic, health status and the descriptive analysis of the six outcome variables for the overall sample as well as the samples Gusu (urban) and Jinhu (rural) separately. The sample included 463 adult participants in the urban area and 462 in the rural area. The gender distribution was almost balanced, and 44.1% were over 60 years old. Participants in the urban area had higher socio-economic status in terms of education, employment and income. In the rural sample 27.9% of participants had never completed primary school education, while this was only 2.6% in the urban sample. The average income per capita of the households in the urban sample was almost twice that in the rural sample. Over 95% of the sample were covered by public health insurance, therefore we could not explore the effects of having no public health insurance on health care utilization. Over 70% of urban sample were covered by UEBMI while in the rural sample 87.5% were enrolled in the NRCMS. As for NCD status, 45.7% of the sampled population had at least one type of NCD, and this rate was slightly higher in the rural area. Descriptive analysis on the six outcome variables showed that the rural sample had more self-reported emergent illness episodes, used both more informal and formal health care services, had higher OOP health expenditure and higher likelihood of incurring CHE .

### Factors associated with health care needs

Table 3 shows the association between perceived health care needs and a series of demographic, health status and socio-economic factors, using the ZINB model. Process 1 of the model showed that, after adjusting for other covariates, people with rural residence were much more likely to be at risk of reporting emergent illness as compared to their urban counterparts (OR = 0.02, 95% CI: 0.00, 0.26). Having any NCD also increased the probability of such risk (OR = 0.33, 95% CI: 0.11, 0.91). Process 2 of the model shows that, after adjusting for other factors, having a NCD was in addition associated with reporting more emergent illness episodes (IRR = 1.39, 95%CI: 1.02, 1.89). People enrolled in NRCMS/URBMI also tended to report more emergent illness episodes compared to those enrolled in UEBM, (IRR = 1.67, 95% CI: 1.03, 2.71). Education level seemed negatively associated with the number of self-reported emergent illness episodes, and the association was almost significant for those with highest education level (senior high school and above).

**Table 3:** Regression analysis of factors associated with self-reported emergent illness episodes using ZINB model

	Process 1		Process 2	
	OR	95% CI	IRR	95% CI
Age				
< 30	ref.		ref.	
30–59	0.24	0.03, 1.81	0.76	0.30, 1.88
> =60	0.41	0.05, 2.84	0.73	0.30, 1.78
Male	0.88	0.31, 2.50	0.91	0.68, 1.24
Rural residence	0.02	0.00, 0.26	0.73	0.41, 1.29
Married	0.76	0.28, 2.02	0.93	0.65, 1.32
Education Level				
no education	ref.		ref.	
primary and junior high	1.36	0.18, 9.78	0.86	0.64, 1.16
senior high school and above	0.76	0.07, 7.22	0.6	0.36, 1.01
Employed	1.17	0.36, 3.77	1.07	0.79, 1.43
Insurance				
UEBMI	ref.		ref.	
NRCMS/URBMI	1.75	0.66, 4.65	1.67	1.03, 2.71
Income level				
poorest 33.3%	ref.		ref.	
middle 33.3%	0.92	0.19, 4.29	0.94	0.67, 1.32
richest 33.3%	0.75	0.19, 2.86	0.85	0.58, 1.23
With NCD	0.33	0.11, 0.91	1.39	1.02, 1.89

(*OR* odds ratio, *IRR* incident rate ratio. Process 1 modeled the likelihood of not being at risk of reporting self-reported illness, process 2 modeled the total number of self-reported emergent illness episodes given that one is at risk. The sample size is the same as described in Table 2. All estimates were adjusted.)

### Factors associated with use of self-treatment, outpatient and inpatient service

Table 4 shows the analyses of the numbers of self-treatments, outpatient service use and inpatient service use for a series of demographic, health status and socio-economic factors, using different regression models. Multivariable NB regression of self-treatment on these factors showed that older age, rural residence and having NCD were significantly associated with increased use of self-treatment, and the effect was particularly strong for rural residence (IRR = 6.07, 95% CI: 2.86, 12.88). As for outpatient service use, multivariable regression analysis using the ZINB model showed that rural residence was associated with much higher probability of being at risk of using outpatient services (i.e. using any of these services) compared to urban residence (OR = 0.02, 95%CI: 0.00, 0.10). Conversely, after adjusting for other covariates, enrollment in NRCMS/RBMI significantly decreased the probability of using any outpatient service compared to UEBMI (OR = 13.29, 95% CI: 1.34, 132.24), which means NRCMS/RBMI may discourage outpatient service use. Nevertheless, for those who were at risk of using outpatient service, NRCMS/RBMI was significantly associated with more use (IRR = 2.75, 95% CI: 1.13, 6.72). Multivariable logit regression of inpatient service use showed that rural residence and having NCD were associated with higher probability of using inpatient service (*P* < 0.05), while men were less likely to use inpatient service than female (*P* < 0.05).

**Table 4:** Regression analysis of factors associated with self-treatment, outpatient service and inpatient service use

	Self-treatment		Outpatient service use				Inpatient service use	
	NB		ZINB-proc1		ZINB-proc2		logit	
	IRR	95% CI	OR	95% CI	IRR	95% CI	OR	95% CI
Age								
< 30	ref.		ref.				ref.	
30–59	2.41	1.24, 4.69	0.64	0.13, 3.07	0.75	0.32, 1.75	0.34	0.10, 1.15
> =60	2.27	1.10, 4.68	0.76	0.10, 5.93	0.59	0.25, 1.40	1.11	0.31, 3.97
Male	0.94	0.67, 1.30	1.66	0.48, 5.76	0.98	0.71, 1.37	0.37	0.18, 0.77
Rural residence	6.07	2.86, 12.88	0.02	0.00, 0.10	0.56	0.17, 1.85	3.56	1.60, 7.93
Married	0.87	0.57, 1.31	0.59	0.08, 4.13	0.86	0.55, 1.35	1.85	0.75, 4.52
Education Level								
no education	ref.		ref.				ref.	
primary and junior high school	1.09	0.77, 1.53	0.5	0.05, 4.97	0.71	0.47, 1.08	1.27	0.54, 2.99
senior high school above	0.87	0.49, 1.54	1.3	0.04, 39.13	0.71	0.33, 1.52	1.7	0.53, 5.57
Employed	0.91	0.62, 1.33	1.45	0.28, 7.57	1.07	0.72, 1.58	0.61	0.28, 1.30
Insurance								
UEBMI	ref.		ref.				ref.	
NRCMS/URBMI	1.48	0.75, 2.91	13.29	1.34, 132.24	2.75	1.13, 6.72	1.1	0.46, 2.62
Income level								
poorest 33.3%	ref.		ref.				ref.	
middle 33.3%	0.89	0.61, 1.30	0.26	0.02, 2.86	0.79	0.56, 1.12	1.52	0.69, 3.33
richest 33.3%	0.61	0.34, 1.09	0.21	0.00, 13.65	0.71	0.42, 1.20	1.07	0.46, 2.50
With NCD	1.49	1.12, 1.99	0.06	0.00, 4.97	1.21	0.80, 1.85	2.65	1.41, 4.95

(*OR* odds ratio, *IRR* incident rate ratio, *Proc* Process. Process 1 of ZINB modeled the likelihood of not being at the risk of using outpatient service, and process 2 modeled the total times of outpatient service use given that one is at that risk. The sample size is the same as described in Table 2. All estimates were adjusted.)

### Out-of-pocket (OOP) payment and financial burden across income groups

Table 5 shows the results of regression analyses of factors associated with OOP health expenditure using a two-part model combining logit regression and GLM, as well as factors associated with CHE using a logit model. Similar to the results of the analysis on inpatient service use, NCD and rural residence were significantly associated with higher probability of incurring medical expenditure and CHE (*P* < 0.001) after adjusting for other covariates. For those who had out-of-pocket health expenditure, men tended to spend less than women, and men were also less likely to incur catastrophic expenditure (*P* < 0.05). People in NRCMS/RBMI were also twice likely to incur CHE as those enrolled in UEBMI (OR = 2.02, 95% CI: 1.10, 3.73), after adjusting for other variables.

**Table 5:** regression analysis of factors associated out-of-pocket health expenditure and CHE

	OOP health expenditure				Catastrophic health expenditure	
	part1-logit		part2-GLM		logit	
	OR	95% CI	Coef.	95% CI	OR.	95% CI
Age						
< 30	ref.				ref.	
30–59	1.46	0.73, 2.92	57.7	− 1810.2, 1925.7	1.01	0.40, 2.59
> =60	1.07	0.51, 2.21	1898.1	− 722.9, 4519.0	1.13	0.44, 2.94
Male	0.8	0.58, 1.11	− 2207.4	− 4337.5, −77.3	0.58	0.38, 0.89
Rural residence	6.6	3.95, 11.03	1094.2	− 802.5, 2991.0	2.92	1.61, 5.30
Married	1.16	0.72, 1.86	595	− 620.0, 1810.0	0.94	0.53, 1.67
Education Level						
no education	ref.				ref.	
primary and junior high	0.68	0.42, 1.11	1788.4	− 634.9, 4211.7	0.97	0.57, 1.64
senior high school and above	0.62	0.32, 1.18	2199.7	− 761.7, 5161.1	0.73	0.34, 1.54
Employed	0.9	0.58, 1.39	712.9	− 1453.9, 2879.8	0.61	0.37, 1.02
Insurance						
UEBMI	ref.				ref.	
NRCMS/URBMI	1.33	0.8, 2.19	238.5	− 640.6, 1117.7	2.02	1.10, 3.73
Income level						
poorest 33.3%	ref.				ref.	
middle 33.3%	1.06	0.69, 1.61	− 237.4	− 1661.7, 1186.8	0.72	0.43, 1.20
richest 33.3%	1.09	0.69, 1.74	877.2	− 2167.2, 3921.6	0.57	0.31, 1.05
With NCD	1.99	1.42, 2.78	212.8	− 1245.0, 1670.6	2.97	1.93, 4.58

(Part 1 of the two-part model used logit regression to estimate the likelihood of incurring OOP health expenditure, and part 2 used GLM to model the amount of OOP health expenditure if occurred. All estimates were adjusted.)

### Stratified analysis on the rural and urban sample

We further explored the effects of demographic and SES factors on these outcomes of interests for urban and rural population separately. Gender played a role in the rural but not in the urban area. Compared to women, men in the rural area tended to report fewer emergent illnesses, use less inpatient and outpatient services, and thus less often incurred catastrophic expenditure. It is also noticeable that for the rural sample, people enrolled in NRCMS/URBMI were more likely to incur CHE compared to those enrolled in UEBMI, and being in the richest tertile also decreased the likelihood of incurring CHE. Nevertheless, insurance category and income were not significantly associated with the possibility of incurring CHE in the urban sample, and only NCD status seemed to have an effect on CHE (*P* < 0.05) (see Additional file 2: Tables S2-S6).

## Discussion

Findings from this study revealed a clear rural-urban distinction: the rural population tended to have more perceived health care needs, had a higher probability of using both informal (self-treatment) and formal (outpatient and inpatient) healthcare services, and had more OOP health expenditure and a higher likelihood of incurring catastrophic expenditure after controlling for other factors. The rural-urban difference in perceived health care needs may be due to unobserved disparities in health status, for example, healthier people are more likely to move to urban areas to seek job opportunities. In our study situated in a developed region, the rural population have access to care upon need, non-need factors such as income seemed to have no effect on health care utilization, and insurance type only had an impact on outpatient service use. Nevertheless, while the expansion of insurance coverage, mainly NRCMS/URBMI, and the investment in health care infrastructure have been narrowing the gaps in service use, people in rural areas are still facing a higher financial burden of treatment.

Besides the rural-urban difference, people enrolled in UEBMI were less likely to incur catastrophic expenditures, and our separate analysis of the rural and urban sample showed that this protective effect is significant for the rural population, but no for the urban population. While previous studies have revealed that current benefit packages of NRCMS are not sufficient to protect people from catastrophic spending [4, 32], our study also suggests that we may need to improve the coverage range and reimbursement rate of NRCMS/URBMI to reduce the possibility of catastrophic expenditure, which is particularly urgent when more poor people start to seek care. We also noticed that while enrollment in NRCMS/RBMI indicates lower probability of using any outpatient care, it was associated with increased numbers of visits for those who were at risk of using outpatient service. This finding suggests that as NRCMS/URBMI provides little coverage for outpatient services, people may delay care seeking until the disease is serious, which may in turn lead to higher expenditure for treatment.

As the health care reform in China continues, NRCMS is being, or has been, integrated with URBMI in many regions. Several studies on this integration show that it narrowed the rural-urban gaps in inpatient benefit, improved the quality of health care and reduced the health care expenditure of the rural population [33, 34]. The integration of all the three public insurances has just started to be piloted in some cities [35]. Although some studies have revealed that such integration would encounter administrative and technical challenges [36, 37], it is still regarded by many researchers as a critical way of reducing inequity across insurance schemes and regions [9, 37, 38]. In our study we emphasize that there is a need to reduce the gaps in benefit packages for UEBMI and NRCMS/URBMI, in view of the increasing health care demands from the rural population.

In this study we investigated and identified the inequity in health care needs, service use and financing between rural and urban population, as well as across different types of public insurance. Nevertheless, this study also has several limitations. As mentioned above, it was conducted in the most developed eastern area of China where in 2017 less than 0.8% of the rural population still lived in absolute poverty [39]. Findings from this study may underestimate the level of inequity in health care with regards to the overall situation in China, as poorer people in this area were still able to access inpatient care despite of the high OOP medical expenditure. External validation of the results was also not possible as we only have data in Jiangsu. The short monitoring period and changes in the frequency of follow-up surveys also restricted us in observing seasonal changes in health care needs or service use. Besides, CHE is a household-level variable and we realize that when we use individual regressors as proxy for their household-level equivalents, we introduced measurement error ‘on the right-hand side’, leading to attenuation bias. On the other hand, the fact that we included all adults from a household in our ample tends to neutralize this bias. In light of these findings and limitations, future research may increase the number of study sites and extend the length of monitoring to get a more complete understanding of the equity issues in health care across regions in China at different developmental stages. Besides, the reason why men in rural area made less use of inpatient services remains unclear, and deserves further investigation.

## Conclusion

The rural population in Jiangsu perceived more health care needs and had a higher probability of using both informal and formal health care services than the urban population. Rural population also had higher OOP health expenditure, and NRCMS /URBMI provided less sufficient protection from catastrophic expenditure as compared to UEBMI. While the expansion of the coverage in NRCMS/URBMI has narrowed the gaps in health care utilization, the inequities in health care financing may be further addressed through improving the benefit packages of NRCMS/URBMI.

## Supplementary information

**Additional file 1:** **Questionnaire** Survey questionnaire for this research project.

**Additional file 2. **Results of the model comparison using the Akaike Information Criterion (AIC), the Bayesian Information Criterion (BIC) and the Vuong’s closeness test, and the stratified analysis on the rural and urban sample. **Table S1.** The AIC, BIC and results of Vuong test of different regression models for each outcome variable **Table S2.** Regression analysis of factors associated with self-reported emergent illness using NB model: rural vs urban. **Table S3.** Regression analysis of factors associated with the total times of conducting self-treatment using NB model: rural vs urban. **Table S4.** Regression analysis of factors associated with outpatient service use using NB model: urban vs. rural. **Table S5.** Regression analysis of factors associated with inpatient service use using logit model: urban vs. rural. **Table S6.** Regression analysis of factors associated with OOP health expenditure using a two-part model combining logit regression and GLM: urban vs. rural. **Table S7.** Logit regression analysis of factors associated with the likelihood of incurring CHE: urban vs. rural.

## Data Availability

The datasets generated and/or analyzed during the current study are not publicly available due to the fact that the data are owned by the National Health Commission China. The data could be available after obtaining consent from the National Health Commission China.

## Abbreviations

AIC: Akaike Information Criterion
ALP: Affordability Ladder Program
BIC: Bayesian Information Criterion
CHE: Catastrophic Health Expenditure
CI: Confidence Interval
GLM: Generalized Linear Model
NB: Negative Binomial
NCD: Non-communicable disease
NRCMS: New Rural Cooperative Medical Scheme
OOP: Out-of-pocket
SES: Socio-economic Status
UEBMI: Urban Employee Basic Medical Insurance
URBMI: Urban Resident Basic Medical Insurance
ZINB: Zero-inflated Negative Binomial
ZIP: Zero-inflated Poisson

## References

[1] Zhang X, Xiong Y, Ye J, Deng Z, Zhang X. Analysis of government investment in primary healthcare institutions to promote equity during the three-year health reform program in China. BMC Health Serv Res. 2013. 10.1186/1472-6963-13-114 PMC3614483 23530658

[2] Li L, Fu H (2017). China’s health care system reform: Progress and prospects. Int J Health Plann Manag [Internet].

[3] Chen G, Liu GG, Xu F (2014). The impact of the urban resident basic medical insurance on health services utilisation in China. Pharmacoecon.

[4] Yang W (2015). Catastrophic Outpatient Health Payments and Health Payment-induced Poverty under China’s New Rural Cooperative Medical Scheme. Appl Econ Perspect Policy [Internet].

[5] Sun J, Deng S, Xiong X, Tang S (2014). Equity in access to healthcare among the urban elderly in China: does health insurance matter?. Int J Health Plann Manag.

[6] Liu GG, Zhao Z, Cai R, Yamada T, Yamada T (2002). Equity in health care access to: assessing the urban health insurance reform in China. Soc Sci Med.

[7] X. L, S. T, B. Y, N.K. P, F. Y, D.D. T, et al. Can rural health insurance improve equity in health care utilization? A comparison between China and Vietnam. Int J Equity Health [Internet]. 2012;11(1):1–9. Available from: http://ovidsp.ovid.com/ovidweb.cgi?T=JS&PAGE=reference&D=emed10&NEWS=N&AN=2012234220 10.1186/1475-9276-11-10 PMC3334712 22376290

[8] Zhou Z, Su Y, Gao J, Campbell B, Zhu Z, Xu L (2013). Assessing equity of healthcare utilization in rural China: results from nationally representative surveys from 1993 to 2008. Int J Equity Health [Internet].

[9] World Bank. The path to integrated insurance system in China (Vol. 2) : Main report (English). China health policy notes ; no. 3. Washington, DC: World Bank. 2010. Available from: http://documents.worldbank.org/curated/en/926821468024660940/Main-report.

[10] Tang SL, Meng QY, Chen L, Bekedam H, Evans T, Whitehead M (2008). Health system reform in China 1 tackling the challenges to health equity in China. Lancet.

[11] Hu S, Tang S, Liu Y, Zhao Y, Escobar ML, de Ferranti D (2008). Reform of how health care is paid for in China: challenges and opportunities. Lancet [Internet].

[12] Chen M, Palmer AJ, Si L (2017). Improving equity in health care financing in China during the progression towards universal health coverage. BMC Health Serv Res.

[13] Pan J, Tian S, Zhou Q, Han W (2016). Benefit distribution of social health insurance: evidence from china’s urban resident basic medical insurance. Health Policy Plan.

[14] Minchao J (2011). Health Insurance in Urban China: disparity between peasant workers and legal urban residents. Int J Health Wellness [Internet].

[15] Yang W. China’s new cooperative medical scheme and equity in access to health care: evidence from a longitudinal household survey. Int J Equity Health [Internet]. 2013;12:20. Available from: http://www.pubmedcentral.nih.gov/articlerender.fcgi?artid=3616826&tool=pmcentrez&rendertype=abstract 10.1186/1475-9276-12-20 PMC3616826 23522336

[16] Zhang X, Dupre ME, Qiu L, Zhou W, Zhao Y, Gu D (2017). Urban-rural differences in the association between access to healthcare and health outcomes among older adults in China. BMC Geriatr.

[17] Fu Rong, Wang Yupeng, Bao Han, Wang Zhiqiang, Li Yongquan, Su Shaofei, Liu Meina (2014). Trend of Urban-Rural Disparities in Hospital Admissions and Medical Expenditure in China from 2003 to 2011. PLoS ONE.

[18] Li J, Shi L, Liang H, Ding G, Xu L (2018). Urban-rural disparities in health care utilization among Chinese adults from 1993 to 2011. BMC Health Serv Res.

[19] Wang L, Wang A, Zhou D, FitzGerald G, Ye D, Jiang Q (2016). An empirical analysis of rural-urban differences in out-of-pocket health expenditures in a low-income society of China. PLoS One.

[20] Cai J, Coyte PC, Zhao H (2017). Decomposing the causes of socioeconomic-related health inequality among urban and rural populations in China: a new decomposition approach. Int J Equity Health.

[21] Yeager RD, Yeager RD (2019). Micropolitics in Rural Africa Linked references are available on JSTOR for this article. Micropolitics Rural Afr.

[22] Dahlgren Göran, Whitehead Margaret (2007). A Framework for Assessing Health Systems from the Public's Perspective: The Alps Approach. International Journal of Health Services.

[23] Gilson L, McIntyre D (2007). Post-apartheid challenges: household access and use of health care in South Africa. Int J Health Serv [Internet].

[24] Iyer A, Sen G, George A (2007). The dynamics of gender and class in access to health care: evidence from rural Karnataka, India. Int J Health Serv [Internet]..

[25] Luong DH, Tang S, Zhang T, Whitehead M (2007). Vietnam during economic transition: a tracer study of health service access and affordability. Int J Health Serv [internet].

[26] Jiangsu Government Report 2016 [Internet]. Available from: http://cn.chinagate.cn/reports/2016-03/05/content_37944056.htm

[27] Culyer AJ, Wagstaff A (1993). Equity and equality in health and health care. J Health Econ.

[28] Janicke DM, Finney JW, Riley AW. Children ’ s Health Care Use A Prospective Investigation of Factors Related to Care-Seeking. Med Care. 2018;39(9):990–1001 Riley Published by : Lippincott Williams & Wilkins Stable. https://www.jstor.org/stable/3767778. 10.1097/00005650-200109000-00009 11502956

[29] Ranson MK (2002). Reduction of catastrophic health care expenditures by a community-based health insurance scheme in Gujarat, India: Current experiences and challenges [Internet].

[30] Neelon B, O’Malley AJ, Smith VA (2016). Modeling zero-modified count and semicontinuous data in health services research part 1: background and overview. Stat Med.

[31] Neelon B, O’Malley AJ, Smith VA (2016). Modeling zero-modified count and semicontinuous data in health services research part 2: case studies. Stat Med.

[32] Liang Xiaoyun, Guo Hong, Jin Chenggang, Peng Xiaoxia, Zhang Xiulan (2012). The Effect of New Cooperative Medical Scheme on Health Outcomes and Alleviating Catastrophic Health Expenditure in China: A Systematic Review. PLoS ONE.

[33] Liu P, Guo W, Liu H, Hua W, Xiong L (2018). The integration of urban and rural medical insurance to reduce the rural medical burden in China: a case study of a county in Baoji City. BMC Health Serv Res.

[34] Yang X, Chen M, Du J, Wang Z (2018). The inequality of inpatient care net benefit under integration of urban-rural medical insurance systems in China. Int J Equity Health.

[35] Shan L, Zhao M, Ning N, Hao Y, Li Y, Liang L (2018). Dissatisfaction with current integration reforms of health insurance schemes in China: are they a success and what matters?. Health Policy Plan.

[36] Xin W, Ang Z, Xin H, HangHang J (2014). Integration of rural and urban healthcare insurance schemes in China: an empirical research. BMC Health Serv Res [Internet].

[37] Wang HQ, Liu ZH, Zhang YZ, Luo ZJ (2012). Integration of current identity-based district-varied health insurance schemes in China: implications and challenges. Front Med Chin.

[38] Meng Q, Fang H, Liu X, Yuan B, Xu J (2015). Consolidating the social health insurance schemes in China: towards an equitable and efficient health system. Lancet.

[39] Report series on the achievements in socio-ecnomic development 40 years since the reform opening-up policy in China [Internet]. 2018. Available from: http://www.stats.gov.cn/ztjc/ztfx/ggkf40n/201809/t20180903_1620407.html

