# Supplementary material for: Inequity in healthcare needs, health service use and financial burden of medical expenditures in China: results from a consecutive household monitoring study in Jiangsu Province
Source: BMC Health Serv Res. 2019 Dec 16;19:966. doi: 10.1186/s12913-019-4796-4 (PMC6916066; doi:10.1186/s12913-019-4796-4)
Supplement: Supplementary file 1 — Additional file 1: Questionnaire Survey questionnaire for this research project. [file 12913_2019_4796_MOESM1_ESM.docx]

**Additional file 1**

**Survey questionnaire for this research project**

- **Baseline Survey**

Site number |__||__||__| Household ID |__||__|

Surveillance for Use of Healthcare Services among Residents in Jiangsu Province

**Informed Consent Form**

Organizer：National, Jiangsu Provincial, Gusu District and Jinhu County Health and Family Planning Commission

Research institute: Duke Kunshan University

You’re invited to participate in this project. This form provides information for you to decide whether you’d like to take part in it. Please read carefully and ask us any questions you may have (contact person: Mr. Xiaolin Xu, telephone: 0512-36657089 or Mr. Anqi Chen, 025-83620753).

This study aims to investigate the health and disease status, treatment, healthcare service use, medical costs and reimbursement level, and satisfaction of residents (you and all family members living in the same household), in order to provide information for deepening healthcare reforms, optimizing healthcare resources, and meet the health needs of the public. We will conduct one baseline survey through home visits and follow-up surveys every 1 month for 3 months via self-report (fill out the questionnaire by yourself), telephone, or home visits, whatever works best for you. Baseline survey includes information on age, sex, education, and the like and health status and it takes about 15 minutes to complete; follow-up survey includes changes in health status and service use and costs in the past 30 days and it takes about 5-30 minutes to complete depending on your health status and health service uses. In order to ensure survey quality, we will randomly select 5% of participants to conduct a re-survey by a different interviewer. During the follow up, please keep the following if you have any: inpatient and outpatient health record and payment slips, and receipts for medicine.

No other participation will be required for this study and it will not affect your life in other ways, incur risks, or bring benefits besides answering the survey questions. You do not need to pay any fee. We will give you some small gifts valued at about 100 yuan (total) to thank you for your participation. If you decide to take part in this study, your personal information will be kept confidential. We will assign you a number and that number will be connected to your responses to the surveys, rather than your name. Except permitted by yourself, no information will be released to anyone who is not part of the project team. All team members are trained to follow confidentiality protocol during each step of the process.

Your participation is voluntary and your answers will not affect your access to health care or other rights. You can choose not to take part in it or to withdraw from it anytime without any influence on your medical services or other rights.

Interviewer signature：___________________ Date：_____

I have read this informed consent.

I have the opportunity to ask questions and have received satisfactory answers.

I understand that my participation is voluntary.

I can choose not to take part in it or to withdraw from it anytime without any influence on my medical services or other rights.

Name (please print)：___________________ Signature：___________________ Date：_____

| **“The individual identity information materials which have been obtained in statistical surveys shall not be provided，disclosured by any organizations or individuals，and may not be used for the purpose other than statistics.”Article XXV，Chapter III，The Statistics Law of the P.R.China** |  | **Form No. ：National Health Survey No.1**  **Tabulated by ：China National Health and Family Planning Commission**  **Approved by ：National Bureau of Statistics**  **Approval No. ：** |
| --- | --- | --- |

**Household Health Survey Questionnaire**

**Home Address：__________County（county-level city/district）_________Township（community）_________Village（****neighborhood committee） ___________________________（full address）**

**Administrative division code of the county（county-level city/district）□□□□□□**

**Code of township（community）□ Code of village（neighborhood committee）□ Code of household□□**

**Name of householder：____________ Contact number：**

**Date of commence:**  **/** (**dd**/**mm)** **2013** **Time Begin ____:____**

**Date of completion:** **/** (**dd**/**mm)** **2013 Time End ____:____**

**Signature of interviewer：**

**Date of Verification:** **/** (**dd**/**mm) 2013**  **Signature of supervisor：**

**Form 1: Questionnaire on general household information**

**This form should be filled out by the person in the household who is most knowledgeable about the financial, expenditure and other related condition of the household**

| **No.** | **Question & Options** | **Answers** |
| --- | --- | --- |
|  | How many people lived in this household in the last six months (including adults and children living and dining together)？ |  |
|  | Which type of health institution will you and your family choose when you get sick：  (1) Village Clinic/Community Health Station (2) Township Health Center /Community Health Center （3）County-level secondary public hospital (4) Prefecture-level tertiary public hospital or above (5) Private hospital |  |
|  | How long does it take to go from your house to the above institution (minutes)?  **(In the most available and fastest way, such as travel by vehicle or on foot)** |  |
|  | How much was the income of your family in the previous year (in terms of RMB)? **(Urban residents: cash income; rural residents: net income)** |  |
|  | How much was living consumption expenditure of your family in the previous year (in terms of RMB)? |  |
| a | Of this total, how much was spent on medicine, medical services and other medical products (in terms of RMB)? |  |

**Form 2: Questionnaire on personal information for household** **member**

| **A. Personal information** | | | | | | | |
| --- | --- | --- | --- | --- | --- | --- | --- |
| **Reference No. of the household informant (01 represents the householder, the others follow the sequence happened during the survey)** | | 01  Householder | 02 | 03 | 04 | 05 | 06 |
|  | The informant’s relationship to householder is：  ⑴Himself/Herself ⑵Spouse ⑶Son/Daughter 　⑷Son/Daughter-in-law ⑸Parent ⑹Parent-in-law ⑺Grandparent ⑻Grandchildren ⑼Brother /Sister (10) Other relative |  |  |  |  |  |  |
|  | Contribution to the family income: (1)major (2) minor (3)No |  |  |  |  |  |  |
|  | （For the interviewer）The following questions will be answered by：⑴The informant himself/herself  ⑵Other member of the household（proxy interview） |  |  |  |  |  |  |
|  | Category of registered permanent residence：  ⑴Agricultural ⑵Non-agricultural |  |  |  |  |  |  |
|  | Sex：⑴Male ⑵Female |  |  |  |  |  |  |
|  | Which year and month were you born in? |  |  |  |  |  |  |
|  | Which medical insurance(s) have you been enrolled in?  (1) Urban Employee Basic Medical Insurance  (2) Urban Resident Basic Medical Insurance  (3) New Cooperative Medical Schemes for rural residents  (4) Commercial health insurance  (5) Other health insurance  (6) No insurance coverage |  |  |  |  |  |  |
|  | Are you a receiver of government medical relief?  (1)Yes (2) No (3)No idea |  |  |  |  |  |  |
|  | Are you eligible for the outpatient service reimbursement program for special disease/serious disease?  (1)Yes (2)No |  |  |  |  |  |  |
|  | Marital status: (1)Never married (2) Currently Married  (3) Widowed (4) Divorced (5)Other |  |  |  |  |  |  |
|  | What is the highest level of education that you have completed?  ⑴No formal schooling ⑵Primary school ⑶Secondary school  ⑷High school/Polytechnic school/technical secondary school  (5)Junior college (6)University and above |  |  |  |  |  |  |
|  | What is your employment status now:  (1)Employed (2)Retired (3)Student  (4)Jobless (5)Not working |  |  |  |  |  |  |
| **B. Inpatient service use in the past year** | | | | | | | |
|  | During the past 12 months, have you been in hospital as an inpatient for any disease, injury, physical check-up or childbirth? (1)Yes (2)No（Skip to Question 14） |  |  |  |  |  |  |
| a | How many times have you been hospitalized? |  |  |  |  |  |  |
| b | The total out-of-pocket expenditure for inpatient service use (RMB) |  |  |  |  |  |  |
|  | During the past 12 months, was there any time when you really needed to be hospitalized following a recommendation from a doctor, but did not?  (1)Yes (2)No (skip to Question 15) |  |  |  |  |  |  |
| a | What was the main reason for not being hospitalized for the last time?  (1)Don’t think it’s necessary (2)Don’t have effective treatment (3)Financial difficulty (4)The service of hospital is poor (5) Have no time to see a doctor (6) Ward beds tension  (7) Hospital too far away (8) other reasons |  |  |  |  |  |  |
|  | During the past 12 months, was there any time when you think you needed to be hospitalized, but did not?  (1)Yes (2)No (skip to Question 16) |  |  |  |  |  |  |
| a | What was the main reason for not being hospitalized for the last time?  (1)Don’t think it’s necessary (2)Don’t have effective treatment (3)Financial difficulty (4)The service of hospital is poor (5) Have no time to see a doctor (6) Ward beds tension  (7) Hospital too far away (8) other reasons |  |  |  |  |  |  |
| **C. Health state descriptions** | |  |  |  |  |  |  |
|  | Mobility：  ⑴I have no problems in walking about  ⑵I have some problems in walking about  ⑶I am confined to bed |  |  |  |  |  |  |
|  | Self-Care：  ⑴I have no problems with self-care  ⑵I have some problems washing or dressing myself  ⑶I am unable to wash or dress myself |  |  |  |  |  |  |
|  | Usual Activities (e.g. work, study, housework, family or  leisure activities)：  ⑴I have no problems with performing my usual activities  ⑵I have some problems with performing my usual activities  ⑶I am unable to perform my usual activities |  |  |  |  |  |  |
|  | Pain/Discomfort：  ⑴I have no pain or discomfort  ⑵I have moderate pain or discomfort  ⑶I have extreme pain or discomfort |  |  |  |  |  |  |
|  | Anxiety/Depression：  ⑴I am not anxious or depressed  ⑵I am moderately anxious or depressed  ⑶I am extremely anxious or depressed |  |  |  |  |  |  |
|  | Please indicate on the scale how good or bad your own health is today, in your opinion.  ├---┼—-┼—-┼—-┼—-┼—-┼—-┼—-┼—-┼—-┤  0 10 20 　30 　40 　50 60 70 80 90 100  Worst imaginable health state Best imaginable health state |  |  |  |  |  |  |
| **D. Chronic disease condition** | |  |  |  |  |  |  |
|  | Have you ever been diagnosed with hypertension by a medical doctor?   - 1. Yes ⑵No（Skip to 23） |  |  |  |  |  |  |
| a | Year of diagnosis: |  |  |  |  |  |  |
| b | Blood pressure level at diagnosis:  (1) Level 1 (SBP 140-159 and/or DBP 90-99)  (2) Level 2 (SBP 160-179 and/or DBP 100-109)  (3) Level 3 (SBP>=180 and/or DBP>=110)  (4)Unknown |  |  |  |  |  |  |
|  | Have you ever been diagnosed with diabetes by a medical doctor?   - 1. Yes ⑵No（Skip to 24） |  |  |  |  |  |  |
| a | Year of diagnosis |  |  |  |  |  |  |
| b | Fasting blood glucose level at diagnosis (mmol/L) |  |  |  |  |  |  |
|  | Have you had any other chronic disease that diagnosed by a medical doctor (multiple choices)?  (1)Tumor (2)Cerebrovascular diseases (3) Cardiovascular diseases (4)Chronic respiratory diseases (5)Degenerative disk disease (6) Rheumatoid arthritis (7)Other _________ |  |  |  |  |  |  |

- **Follow-up Survey**

***The following questions to be asked with those household members who had newly-occurred or continuous emergent disease or injuries. One form for each member. If one member had more than 1 types of disease/injury, on column for each disease/injury***

**Form 3: Questionnaire for the household member with emergent diseases or injuries in the past two weeks**

**Reference No. of the household informant_______Name__________**

| **NO.** | **Question** | **Answer** |
| --- | --- | --- |
|  | Do you know what was your disease or injury? (Disease or injury name) |  |
|  | Your disease or injury | code |
|  | When did you start to feel sick?  ⑴Within the past two weeks  ⑵Acute disease prior to the past two weeks |  |
|  | How did you feel about your disease/injury?  (1) Minor (2) Mild (3) Serious |  |
|  | How many days did the disease or injury last in the previous two weeks (limit to 14 days)? |  |
|  | During the past two weeks, how many days did you stay in bed due to the disease or injury (limit to 14 days) ?（If no, write “0”） |  |
|  | During the past two weeks, how many days did you absence from work due to the disease or injury (limit to 14 days) ?（If no, write “0”） |  |
|  | During the past two weeks, how many days did you absence from school due to the disease or injury (limit to 14 days) ?（If no, write “0”） |  |
|  | Have you tried to understand your disease/injury through the following channels (multiple choices)?  (1) Telephone or face-to-face consultation (2) Internet or cellphone consultation (3) Read books (4) Past experiences (5) Other_________ |  |
|  | During the past two weeks, how did you cope with the disease/injury?  (1) No measures taken (continue with a) (2)Self-treatment only  (3) Seeing a doctor after ineffective self-treatment [continue with 11 if (2)or(3) was selected]  (4) Directly went to see a doctor (skip to other forms) |  |
| a | If no measures taken, what was the major reason(s)?  (1) Self-rate just a minor disease (2) Have no time to see a doctor  (3) Inconvenient visiting (4) Traffic inconvenience (5) Financial difficulty  (6) Feel that there was no effective treatment (7) Other reasons  (This form completed, please continue with other forms) |  |
|  | During the past two weeks, how much did you pay out-of-pocket for self-treatment?  （It doesn’t include reimbursement and cost in individual account of social medical insurance） |  |
|  | In case of self-medication, had you ever chosen medicine？  (1)Yes (2) No (Skip to other forms) |  |
| a | The type of medicine used (multiple choices):  (1) Western medicine (2)TCM (3) Chinese patent drug (4) Other |  |
|  | In case of self-medication, where did you get the medicine？（Choose two answers at most）  (1) Village Clinic/Community Health Station (2) Township Health Center /Community Health Center （3）County-level secondary public hospital (4) Prefecture-level tertiary public hospital or above (5) Drug store (6)Online shopping (7)Other sources |  |
| a | In case of bought in last two weeks，how much did you spent on the medicine? |  |
| b | Of the total, how much did you pay out-of-pocket (cash)? |  |
| c | Of the total, how much did you pay using insurance card? |  |
|  | During the past two weeks, did you use TCM medical services? ⑴Yes ⑵No (skip to other forms) |  |
| a | How much did you pay out-of-pocket on TCM medical services? |  |

***The following questions to be asked with those household members who used outpatient service (including outpatient emergency treatment). One form for each visit***

**Form 4: Questionnaire for the household member who used outpatient services (including outpatient emergency treatment) in the past two weeks**

**Reference No. of the household informant_______Name__________**

| **NO.** | **Question** | **Answer** |
| --- | --- | --- |
|  | Major diagnosis from the doctor  Other diagnosis | code  code |
|  | Name of the health agency |  |
|  | The reason(s) why you choose this health agency:  (1)low cost (2)convenient transportation (3)acquaintances in that health agency  (4)good and reliable skills (5)Treatment cost could be covered by health insurance (6)With doctors from upper level hospital (7)Short waiting time  (8)Other reasons |  |
|  | Transportation to this health agency:  (1) walk (2) bicycle or other human-powered vehicles (3) motorbike  (4) private care or taxi (5)bus/tram or other public transportation (6) other |  |
|  | How long did it take to travel to the health agency (minutes)? |  |
|  | Have you received intravenous infusion treatment for this outpatient visit?  (1) Yes (2)No |  |
|  | The total cost for this outpatient visit (RMB): |  |
| a | Of the total, how much did you pay out-of-pocket (cash)? |  |
| b | Of the total, how much did you pay using insurance card? |  |
| c | Of the total, how much did you pay through special disease outpatient reimbursement program? |  |
| d | Of the total, how much did you pay through other reimbursement approaches? |  |
|  | What was the major treatment plan the doctor suggested during this visit?  (1) Outpatient treatment in this health agency (skip to question 9)  (2) Transfer to other health agency (skip to question 10)  (3) Hospitalization treatment in this health agency (skip to question 11)  (4) Other_____________(skip to question 12) |  |
|  | Did you follow the advice of outpatient treatment in this health agency?  (1) Yes (2) No  (continue with question 12-13 after this question) |  |
|  | Did you follow the advice of transferring to other health agency?  (1) Yes (2) No |  |
| a | The name of the health agency the doctor suggested that you transfer to |  |
| b | The reason(s) why the doctor suggested that you transfer to other agency:  (1) This agency is not technically capable for treating the disease  (2) Policy related reasons (e.g., the coordinated care system)  (3) Other______________ |  |
| c | If you did not follow the advice, the major reasons were (multiple choices):  (1) Self-rate just a minor disease (2) Financial difficulty  (3) no time (4) Traffic inconvenience (5) inconvenient transfer procedure  (6) Other reasons  (continue with question 12-13 after this question) |  |
|  | Did you follow the advice of inpatient treatment in this agency?  (1) Yes (2) No |  |
| a | If you did not follow the advice of hospitalization, the major reasons were (multiple choices):  (1) Self-rate just a minor disease (2) Financial difficulty  (3) no time (4) Traffic inconvenience (5) no bed available in the agency  (6) Other reasons  (continue with question 12-13 after this question) |  |
|  | In general would you say how satisfied for the visit？  ⑴Satisfied(skip to 111) ⑵Neither satisfied nor dissatisfied ⑶Dissatisfied |  |
| 13 | In case of dissatisfaction, which aspect dissatisfied you most？（Choose three answers at most）  (1) Overcharge (2) Low skills (3) Long waiting  (4) Too much formalities (5) Unreasonable charges  (6) Some unnecessary services were provided(medicine and check-ups)  (7) Bad attitudes from service providers (8) Limited choices of medicine  (9) Poor environment (10) Low quality equipment (11) Other__________ |  |

***The following questions to be asked with those household members who used inpatient service. One form for each hospitalization***

**Form 5: Questionnaire for the household member who used inpatient services in the past two weeks**

**Reference No. of the household informant_______Name__________**

| **NO.** | **Question** | **Answer** |
| --- | --- | --- |
|  | Name of the health agency |  |
|  | Major diagnosis from the doctor | code |
|  | Date of admission DD/MM/YY |  |
|  | Date of discharge DD/MM/YY |  |
|  | Channel of hospitalization:  (1) Directly from the outpatient or emergency department of this agency  (2) Transferred from other hospital |  |
| a | If transferred from other hospital, what services you enjoyed during the transfer process?  (1) Received certificate of transfer (2) Accompanied by a health worker  (3) Assistance in contacting this agency (4) Ambulance  (5) Provide materials such as lab test results and medical records  (6) Other_____________ (7) None |  |
|  | During the hospitalization, did you have an operation? (1)Yes (2) No |  |
|  | During this hospitalization, have you used TCM services?  (1) Hospitalization in TCM hospital ⑵ Hospitalization in TCM department of the integrated hospital ⑶ No TCM |  |
|  | During this hospitalization, did you receive consultation service by doctors from other hospitals? (1)Yes (2) No |  |
|  | Department of hospitalization____________ |  |
|  | Did you encounter any of the situation below during this hospitalization?  (1) Transferred to other department in this agency  (2) Transferred to other agency  (3) None |  |
|  | You were discharged from the hospital because of ：  ⑴ Doctor’s request after you have recovered  ⑵ Doctor’s request before you have recovered  ⑶ Your own request ⑷ Other reasons |  |
| a | If it was doctor’s request before you have recovered , the reason(s) were:  (1) Taking medication at home (2) Limitation on the duration of hospitalization, and got admitted again after discharge  (3) Transferred to lower level health agency  (4) Transferred to higher level health agency  (5) Financial reasons (6) No effective treatment (7) Other reason_____ |  |
| b | If it was your own request, the reason(s) were：  (1)Have been sick for quite some time, but see little hope of recovery  ⑵Self-considered have recovered ⑶Financial difficulty  ⑷Hospital charges were too expensive ⑸Facility of the hospital is poor  ⑹Service of the hospital is poor ⑺Technology of the hospital is poor  ⑻Other reasons |  |
|  | The total cost for this inpatient treatment (RMB): |  |
| a | Of the total, how much did you pay out-of-pocket (cash)? |  |
| b | Of the total, how much did you pay using insurance card? |  |
| c | Of the total, how much did you pay through serious disease t reimbursement program? |  |
| d | Of the total, how much did you pay through other reimbursement approaches? |  |
|  | Transportation fee for this inpatient treatment (accompanies’ costs included): |  |
|  | Accommodation fee for this inpatient treatment (accompanies’ costs included): |  |
|  | Which was the mainly caregiver during your hospitalization?   1. Relatives (2) Professional care giver (3) No |  |
| a | In case of relative(s), the cost for the loss of working time was |  |
| b | In case of professional givers, the cost of employing them was |  |
|  | In general would you say how satisfied for the hospitalization experience?  (1) Very satisfied (2) Satisfied (3) Neither satisfied nor dissatisfied  (4) Dissatisfied (5) Very dissatisfied |  |
|  | In case of dissatisfaction, which aspect dissatisfied you most？（Choose three answers at most）  (1) Overcharge (2) Unreasonable charges (3) Long waiting  (4) Too much formalities (5) Limited choices of medicine  (6) Some unnecessary services were provided (medicine and check-ups)  (7) Bad attitudes from service providers (8) Low skills  (9) Poor environment (10) Low quality equipment (11) Other__________ |  |
|  | After discharge from hospital, have you communicated with the primary health care workers from community health care centers/village health clinics on your hospitalization and your subsequent treatment? (1) Yes (2) No |  |
| a | If yes, what suggestions or health services have the primary health care workers from community health care centers/village health clinics offered?   1. Guidance on recovery (2) Follow-up visit at regular time   (3) Recovery treatment (4) Others |  |
| b | If no communication, the reason(s) was:  (1) Low skills (2) Primary health care workers did not provide such services  (3) Feel unnecessary (4) Others |  |
