# Supplementary material for: Inequity in healthcare needs, health service use and financial burden of medical expenditures in China: results from a consecutive household monitoring study in Jiangsu Province
Source: BMC Health Serv Res. 2019 Dec 16;19:966. doi: 10.1186/s12913-019-4796-4 (PMC6916066; doi:10.1186/s12913-019-4796-4)
Supplement: Supplementary file 2 — Additional file 2. Results of the model comparison using the Akaike Information Criterion (AIC), the Bayesian Information Criterion (BIC) and the Vuong’s closeness test, and the stratified analysis on the rural and urban sample. Table S1. The AIC, BIC and results of Vuong test of different regression models for each outcome variable Table S2. Regression analysis of factors associated with self-reported emergent illness using NB model: rural vs urban. Table S3. Regression analysis of factors associated with the total times of conducting self-treatment using NB model: rural vs urban. Table S4. Regression analysis of factors associated with outpatient service use using NB model: urban vs. rural. Table S5. Regression analysis of factors associated with inpatient service use using logit model: urban vs. rural. Table S6. Regression analysis of factors associated with OOP health expenditure using a two-part model combining logit regression and GLM: urban vs. rural. Table S7. Logit regression analysis of factors associated with the likelihood of incurring CHE: urban vs. rural. [file 12913_2019_4796_MOESM2_ESM.docx]

**Additional file 2**

**Results of the model comparison using the Akaike Information Criterion (AIC), the Bayesian Information Criterion (BIC) and the Vuong’s closeness test, and the stratified analysis on the rural and urban sample**

**Table S1.** The AIC, BIC and results of Vuong test of different regression models for each outcome variable

| **Outcome variable: number of self-reported emergent illness episodes** | | |
| --- | --- | --- |
|  | AIC | BIC |
| Poisson | 2596.1 | 2663.7 |
| Negative binomial | 2336.5 | 2408.9 |
| Zero-inflated poisson | 2379.9 | 2515.1 |
| Zero-inflated negative binomial | 2301.9 | 2441.9 |
| Vuong test of ZINB vs. standard negative binomial: z = 3.70 Pr>z = 0.0001 | | |
| **Outcome variable: total times of self-treatment** | | |
|  | AIC | BIC |
| Poisson | 1375.6 | 1451.0 |
| Negative binomial | 1292.6 | 1365.0 |
| Zero-inflated poisson | 1303.2 | 1438.4 |
| Zero-inflated negative binomial | not concave | |
| **Outcome variable: total times of outpatient visits** | | |
|  | AIC | BIC |
| Poisson | 2200.8 | 2268.4 |
| Negative binomial | 1917.8 | 1990.3 |
| Zero-inflated poisson | 1972.3 | 2107.5 |
| Zero-inflated negative binomial | 1900.7 | 2040.8 |
| Vuong test of ZINB vs. standard negative binomial: z = 3.43 Pr>z = 0.0003 | | |
| **Outcome variable: total OOP health expenditure** | | |
|  | AIC | BIC |
| Glm | 17743.2 | 17810.5 |
| Two-part model: logit+glm | 7978.6 | 8108.5 |

**Table S2.** Regression analysis of factors associated with self-reported emergent illness using NB model: rural vs urban

|  | Rural sample | | Urban sample | |
| --- | --- | --- | --- | --- |
|  | IRR | P>z | IRR | P>z |
| Age |  |  |  |  |
| <30 | ref. |  | ref. |  |
| 30-59 | 1.12 | 0.727 | 1.05 | 0.931 |
| >=60 | 1.04 | 0.902 | 0.77 | 0.680 |
| Male | 0.80 | 0.038 | 1.25 | 0.304 |
| Married | 1.03 | 0.886 | 0.80 | 0.540 |
| Education Level |  |  |  |  |
| no education | ref. |  | ref. |  |
| primary and junior high | 0.94 | 0.627 |  |  |
| senior high school and above | 0.61 | 0.030 | 1.17 | 0.583 |
| Employed | 0.96 | 0.772 | 0.90 | 0.719 |
| Insurance |  |  |  |  |
| UEBMI | ref. |  | ref. |  |
| RBMI | 1.95 | 0.011 | 0.77 | 0.459 |
| Income level |  |  |  |  |
| poorest 33.3% | ref. |  | ref. |  |
| middle 33.3% | 1.03 | 0.820 | 0.74 | 0.525 |
| richest 33.3% | 0.89 | 0.454 | 0.92 | 0.856 |
| With NCD | 1.54 | 0.000 | 2.40 | 0.006 |

**Table S3.** Regression analysis of factors associated with the total times of conducting self-treatment using NB model: rural vs urban

|  | Rural sample | | Urban sample | |
| --- | --- | --- | --- | --- |
|  | IRR | P>z | IRR | P>z |
| Age |  |  |  |  |
| <30 | ref. |  |  |  |
| 30-59 | 1.92 | 0.086 | 5.39 | 0.025 |
| >=60 | 1.81 | 0.148 | 5.13 | 0.064 |
| Male | 0.89 | 0.463 | 1.25 | 0.614 |
| Married | 0.94 | 0.793 | 1.10 | 0.849 |
| Education Level |  |  |  |  |
| no education | ref. |  | ref. |  |
| primary and junior high | 1.06 | 0.768 |  |  |
| senior high school and above | 0.68 | 0.203 | 3.31 | 0.009 |
| Employed | 0.91 | 0.660 | 0.24 | 0.121 |
| Insurance |  |  |  |  |
| UEBMI | ref. |  | ref. |  |
| RBMI | 1.55 | 0.248 | 1.02 | 0.968 |
| Income level |  |  |  |  |
| poorest 33.3% | ref. |  |  |  |
| middle 33.3% | 0.96 | 0.832 | 0.30 | 0.050 |
| richest 33.3% | 0.96 | 0.871 | 0.04 | 0.000 |
| With NCD | 1.56 | 0.005 | 1.25 | 0.676 |

**Table S4.** Regression analysis of factors associated with outpatient service use using NB model: urban vs. rural

|  | Rural sample | |  | Urban sample | |
| --- | --- | --- | --- | --- | --- |
|  | IRR | P>z |  | IRR | P>z |
| Age |  |  |  |  |  |
| <30 | ref. |  |  |  |  |
| 30-59 | -0.151 | 0.689 |  | 0.035 | 0.950 |
| >=60 | -0.370 | 0.362 |  | -0.249 | 0.699 |
| Male | -0.391 | 0.007 |  | 0.190 | 0.403 |
| Married | -0.011 | 0.961 |  | -0.227 | 0.544 |
| Education Level |  |  |  |  |  |
| no education | ref. |  |  | ref. |  |
| primary and junior high | -0.019 | 0.919 |  |  |  |
| senior high school and above | -0.551 | 0.087 |  | -0.012 | 0.966 |
| Employed | -0.106 | 0.600 |  | 0.045 | 0.884 |
| Insurance |  |  |  |  |  |
| UEBMI | ref. |  |  | ref. |  |
| RBMI | 1.005 | 0.017 |  | -0.203 | 0.590 |
| Income level |  |  |  |  |  |
| poorest 33.3% | ref. |  |  | ref. |  |
| middle 33.3% | 0.072 | 0.694 |  | 0.200 | 0.670 |
| richest 33.3% | -0.358 | 0.086 |  | 0.559 | 0.233 |
| With NCD | 0.535 | 0.000 |  | 0.911 | 0.006 |

**Table S5.** Regression analysis of factors associated with inpatient service use using logit model: urban vs. rural

|  | Rural sample | | Urban sample | |
| --- | --- | --- | --- | --- |
|  | Coef. | P>z | Coef. | P>z |
| Age |  |  |  |  |
| <30 | ref. |  | ref. |  |
| 30-59 | 0.86 | 0.689 | 1.04 | 0.950 |
| >=60 | 0.69 | 0.362 | 0.78 | 0.699 |
| Male | 0.68 | 0.007 | 1.21 | 0.403 |
| Married | 0.99 | 0.961 | 0.80 | 0.544 |
| Education Level |  |  |  |  |
| no education | ref. |  | ref. |  |
| primary and junior high | 0.98 | 0.919 |  |  |
| senior high school and above | 0.58 | 0.087 | 0.99 | 0.966 |
| Employed | 0.90 | 0.600 | 1.05 | 0.884 |
| Insurance |  |  |  |  |
| UEBMI | ref. |  | ref. |  |
| RBMI | 2.73 | 0.017 | 0.82 | 0.590 |
| Income level |  |  |  |  |
| poorest 33.3% | ref. |  | ref. |  |
| middle 33.3% | 1.07 | 0.694 | 1.22 | 0.670 |
| richest 33.3% | 0.70 | 0.086 | 1.75 | 0.233 |
| With NCD | 1.71 | 0.000 | 2.49 | 0.006 |

**Table S6.** Regression analysis of factors associated with OOP health expenditure using a two-part model combining logit regression and GLM: urban vs. rural

|  | Rural sample | | | | Urban sample | | | |
| --- | --- | --- | --- | --- | --- | --- | --- | --- |
|  | Logit | | GLM | | Logit | | GLM | |
|  | OR | P>z | Coef. | P>z | OR | P>z | Coef. | P>z |
| Age |  |  |  |  |  |  |  |  |
| <30 | ref. |  |  |  | ref. |  |  |  |
| 30-59 | 1.32 | 0.558 | -614.0 | 0.549 | 0.96 | 0.946 | -1174.6 | 0.055 |
| >=60 | 1.00 | 0.997 | 1202.6 | 0.367 | 0.62 | 0.446 | 639.0 | 0.654 |
| Male | 0.74 | 0.167 | -2258.3 | 0.086 | 0.94 | 0.812 | -1905.9 | 0.269 |
| Married | 1.09 | 0.784 | 647.4 | 0.344 | 1.37 | 0.454 | 288.8 | 0.787 |
| Education Level |  |  |  |  |  |  |  |  |
| no education | ref. |  |  |  | ref. |  |  |  |
| primary and junior  high | 0.76 | 0.331 | 1891.9 | 0.184 |  |  |  |  |
| senior high school  and above | 0.45 | 0.047 | 1113.7 | 0.452 | 1.36 | 0.346 | 2456.0 | 0.184 |
| Employed | 0.89 | 0.673 | 779.8 | 0.550 | 0.74 | 0.456 | -1047.5 | 0.303 |
| Insurance |  |  |  |  |  |  |  |  |
| UEBMI | ref. |  |  |  | ref. |  |  |  |
| RBMI | 1.94 | 0.087 | 43.3 | 0.944 | 0.85 | 0.676 | -15.7 | 0.983 |
| Income level |  |  |  |  |  |  |  |  |
| poorest 33.3% | ref. |  |  |  | ref. |  |  |  |
| middle 33.3% | 1.15 | 0.561 | -228.3 | 0.790 | 0.76 | 0.605 | -1248.3 | 0.334 |
| richest 33.3% | 1.19 | 0.522 | 947.4 | 0.650 | 0.70 | 0.512 | -530.9 | 0.579 |
| With NCD | 1.85 | 0.003 | 174.6 | 0.832 | 2.52 | 0.010 | -7.9 | 0.992 |

**Table S7.** Logit regression analysis of factors associated with the likelihood of incurring CHE: urban vs. rural

|  | Rural sample | | Urban sample | |
| --- | --- | --- | --- | --- |
|  | OR | P>z | OR | P>z |
| Age |  |  |  |  |
| <30 | ref. |  | ref. |  |
| 30-59 | 0.38 | 0.118 | 2.29 | 0.526 |
| >=60 | 0.47 | 0.230 | 1.48 | 0.755 |
| Male | 0.45 | 0.003 | 1.02 | 0.967 |
| Married | 1.14 | 0.696 | 0.53 | 0.197 |
| Education Level |  |  |  |  |
| no education | ref. |  | ref. |  |
| primary and junior high | 1.23 | 0.471 |  |  |
| senior high school and above | 0.21 | 0.006 | 1.62 | 0.340 |
| Employed | 0.67 | 0.180 | 0.49 | 0.368 |
| Insurance |  |  |  |  |
| UEBMI | ref. |  | ref. |  |
| RBMI | 7.30 | 0.033 | 1.89 | 0.359 |
| Income level |  |  |  |  |
| poorest 33.3% | ref. |  | ref. |  |
| middle 33.3% | 0.78 | 0.379 | 0.74 | 0.758 |
| richest 33.3% | 0.27 | 0.010 | 0.89 | 0.908 |
| With NCD | 2.53 | 0.000 | 7.06 | 0.002 |
